# Birth Weight-Dependent Regional Disparities in 28-Day and 1-Year Survival of Preterm Infants: Seoul Capital Area vs. Non-Capital Regions, South Korea, 2002–2021

**DOI:** 10.3390/children13020217

**Published:** 2026-02-02

**Authors:** Ji-Sook Kim, Jong-Yeon Kim, Hyeong-taek Woo

**Affiliations:** 1 Department of Pediatrics, School of Medicine, Kyungpook National University, Daegu 41944, Republic of Korea; 2 Department of Preventive Medicine, School of Medicine, Kyungpook National University, Daegu 41944, Republic of Korea; kom824@knu.ac.kr; 3 Daegu Public Health Policy Institute, Daegu 41940, Republic of Korea; 4 Department of Preventive Medicine, Keimyung University School of Medicine, Daegu 41908, Republic of Korea

**Keywords:** infant, child mortality, low birth weight, health inequities, Korea

## Abstract

**Highlights:**

**What are the main findings?**

- Regional mortality disparities among preterm infants in South Korea increased progressively with decreasing birth weight, with the largest excess mortality observed in extremely low birth weight infants residing outside the Seoul Capital Area.
- For the smallest infants, survival differences were largely attenuated when initial treatment occurred in high-capacity centers, indicating a strong role of regional neonatal care capacity rather than residence alone.

**What are the implications of the main findings?**

- Strengthening high-acuity neonatal intensive care capacity in non-capital regions is critical to reducing preventable mortality among extremely and very low birth weight infants.
- For normal and low birth weight preterm infants, improving timely local stabilization and avoiding unnecessary inter-regional transfers may yield survival benefits without large capital investments.

**Abstract:**

**Background/Objectives**: Regional disparities in neonatal care capacity may have a disproportionate impact on the smallest and most vulnerable infants. In South Korea, where specialized perinatal resources are concentrated in the Seoul Capital Area (Seoul, Gyeonggi, and Incheon), it remains unclear how these disparities vary by birth weight and time since birth. **Methods**: We conducted a nationwide, population-based cohort study of preterm infants (<37 weeks gestation) born between 2002 and 2021 using the Korean National Health Insurance Service database. Residential address at birth classified infants into Seoul Capital Area or regions outside the Seoul Capital Area. We examined 28-day and one-year all-cause mortality using multivariable logistic regression, adjusting for sex, birth weight category, early transfer, medical aid status, maternal age, and antenatal visits. Birth weight-stratified analyses assessed effect modification. Major morbidities were evaluated with multivariable Cox models. **Results**: Among 204,245 preterm infants, those residing outside the Seoul Capital Area had higher adjusted odds of mortality at both 28 days (odds ratio 1.46; 95% confidence interval [CI], 1.30–1.64) and one year (odds ratio 1.25; 95% CI, 1.17–1.34) than those residing in the Seoul Capital Area. Disparities were minimal in infants ≥2500 g but increased progressively in lower birth weight strata, peaking among extremely low birth weight infants (<1000 g) (28-day odds ratio 1.67; 95% CI, 1.40–1.97; one-year odds ratio 1.54; 95% CI, 1.37–1.73). **Conclusions**: Regional survival disparities among preterm infants in South Korea widen with decreasing birth weight, underscoring the need for targeted neonatal care and post-discharge support in underserved regions.

## 1. Introduction

Regionalization, the deliberate organization of health-care services into tiered and geographically defined networks where facilities are classified by capability and connected through structured transfer pathways, aims to match patient risk with the appropriate level of care to improve both effectiveness and efficiency in resource use [1]. In perinatal care, this risk matching is particularly salient because preterm birth is etiologically heterogeneous, arising from spontaneous pathways (e.g., preterm labor, preterm prelabor rupture of membranes, and cervical insufficiency) as well as medically indicated early delivery prompted by complicated pregnancies such as hypertensive disorders and placental disease, and fetal conditions including growth restriction or fetal compromise. These complicated pathways often require uninterrupted high-acuity support and coordinated, timely referral to higher-capability centers; delays or disruptions during inter-regional transfer may therefore disproportionately affect the smallest and most vulnerable infants [2,3]. However, the success of this model relies on a reasonably fair distribution of resources across regions, a condition weakened by the heavy concentration of services in the Seoul Capital Area (SCA) [4,5,6,7]. Prior work underscores the importance of robust, self-sufficient regional networks to achieve optimal survival and developmental outcomes in this high-risk population [8,9].

South Korea’s pronounced centralization, which extends across economic, cultural, and social spheres, is reflected in the health sector, where tertiary hospitals, specialized services, and critical-care capacity remain largely concentrated in the SCA [4,5,6,7]. While such concentration may confer efficiencies, it is associated with inequities in timely access to high-complexity care and with worse outcomes observed in non-SCA regions [10,11,12,13,14]. 

Despite recent policy initiatives and substantial increases in funding aimed at reducing disparities in neonatal care, significant gaps remain, even after the establishment of more regional perinatal centers and the strengthening of formal referral networks [15,16]. Significant regional differences continue to affect the number and capacity of specialized facilities, the availability of experienced clinical personnel, and the deployment of state-of-the-art technologies, all of which contribute to marked variations in both infant mortality and mortality among infants born preterm between SCA and non-SCA regions [17,18,19].

Against this background, we conducted a population-based comparison of 28-day and 1-year survival among infants born preterm (gestational age < 37 weeks) in the SCA versus non-SCA regions during 2002–2021. Our primary objective was to quantify regional disparities in survival. Because the functioning of regionalized perinatal care should matter most for the smallest and most vulnerable infants, we also assessed effect modification by birth weight strata (e.g., <1000 g; 1000–1499 g; 1500–2499 g; ≥2500 g) as an indirect probe of regionalization performance across regions, with the aim of informing policy and resource-allocation strategies.

## 2. Materials and Methods

### 2.1. Data Sources

This study used the Korean National Health Insurance Service (KNHIS) Customized Research Database. The analytic cohort comprised infants born during 2002–2021, and outcomes were followed through June 2023 (maximum follow-up), ensuring sufficient margin to ascertain 1-year mortality. The database comprises records generated during the claims-submission process by care providers after KNHIS beneficiaries received services at medical institutions and has been made available for research purposes. Because the data originate from billing submissions for services rendered in actual clinical settings, they possess the characteristics of real-world data. The database contains 20 years of information on beneficiaries’ insurance eligibility, health-care utilization records—including diagnosis information based on the Korean Standard Classification of Diseases (KCD), which is adapted from the International Classification of Diseases (ICD), as well as fee codes for procedures, surgeries, and medications—and provider characteristics [20].

### 2.2. Study Sample

To construct the customized research database, we first extracted all infants from the KNHIS database who were diagnosed with specific perinatal conditions (KCD codes P00–P96) or congenital malformations, deformations, and chromosomal abnormalities (KCD codes Q00–Q99), establishing our foundational dataset. We then applied the following eligibility criteria to define preterm status: (i) the first preterm code (P07.2, P07.3) had to be assigned within 30 days of a prior health-care utilization record, and (ii) the infant’s age at code assignment was ≤1 year. Using these criteria, we identified the preterm cohort for analysis. Infants born before 1 January 2002, or with missing covariate data, were excluded from the final analytic cohort. 

For all subsequent analyses, we stratified this cohort by region of residence, classifying infants as residing in the Seoul Capital Area (SCA)—a contiguous metropolitan area comprising Seoul (Seoul Special City), Gyeonggi-do (Gyeonggi Province), and Incheon (Incheon Metropolitan City)—or in non-SCA regions (i.e., all other regions of South Korea) [21,22]. We used this SCA/non-SCA stratification to contrast outcomes between the country’s most densely populated and highly urbanized metropolitan region, where tertiary and subspecialty services are concentrated, and the rest of the country, where geographic access and service capacity may differ.

### 2.3. Outcome

We examined three main outcome sets: (1) all-cause mortality at 28 days and 1 year from the date of initial treatment, comparing infants residing in the SCA versus non-SCA regions defined using beneficiaries’ residential address in eligibility records; (2) mortality by residential–treatment region combinations to evaluate potential delays in access to initial treatment among infants whose parents resided outside the SCA, where residential region was defined from parents’ health insurance eligibility records and treatment region was defined by the address of the hospital providing initial care, which was operationalized as the first treating hospital at birth, regardless of any subsequent inter-hospital transfers, comparing 28-day and 1-year mortality across three groups (SCA residence/SCA treatment, non-SCA residence/SCA treatment, and non-SCA residence/non-SCA treatment); and (3) diagnosis-based incidence of major neonatal morbidities—intraventricular hemorrhage (IVH), necrotizing enterocolitis (NEC), late-onset sepsis, retinopathy of prematurity (ROP), and bronchopulmonary dysplasia (BPD)—identified using KCD codes (IVH: P52, I61.5; NEC: P77; sepsis: P36; ROP: H35.1; BPD: P27.1). These comorbidities were selected based on clinical relevance and the prior literature [23]. The occurrence of major neonatal morbidities was compared across regional groups defined by the region of the initial treatment provider (SCA vs. non-SCA).

### 2.4. Statistical Analysis

Descriptive epidemiological analyses were conducted to characterize baseline variables. Statistical significance was assessed using chi-square tests for categorical variables and Student’s *t*-tests for continuous variables. Early transfer was defined as receipt of care at a different facility within one day of initial treatment, and antenatal visits were defined as any prenatal care encounters coded Z34, Z35, or Z36 occurring between 260 and 7 days before the date of initial treatment [24]. To assess regional differences in 28-day and 1-year mortality between the SCA and non-SCA regions, we conducted multivariable logistic regression analyses including sex, categorical birth weight group, transfer status, Medical Aid status, maternal age, and number of antenatal visits as covariates. Gestational age was ascertainable only from claims diagnosis codes and was therefore available only in broad categories (<28 vs. 28–<37 weeks); thus, we primarily stratified and presented results by birth weight categories. Birth weight groups were defined using KCD codes. Although KCD is adapted from ICD-10, it further subdivides P07.1 into 250 g interval subcodes (P07.10–P07.14). Accordingly, we defined normal birth weight (NBW) as ≥2500 g, extremely low birth weight (ELBW) as P07.0 (<1000 g), very low birth weight (VLBW) as P07.10–P07.11 (1000–1499 g), and low birth weight (LBW) as P07.12–P07.14 (1500–2499 g). Analyses were conducted for the entire study period and separately for 2002–2011 and 2012–2021. Furthermore, to evaluate effect modification by birth weight, we conducted stratified analyses across the four birth weight categories. For major comorbidities (IVH, NEC, late-onset sepsis, ROP and BPD), we used multivariable Cox proportional hazards models with the same covariates, as this approach accounts for varying follow-up times in time-to-event outcomes. As a sensitivity analysis for the competing risk of death, we additionally analyzed a composite endpoint in which death was treated as an event together with each morbidity (morbidity-or-death) [25]. All statistical analyses were performed using SAS Enterprise Guide version 7.1 (SAS Institute Inc., Cary, NC, USA).

## 3. Results

### Baseline Characteristics of the Study Population

**Figure 1:**
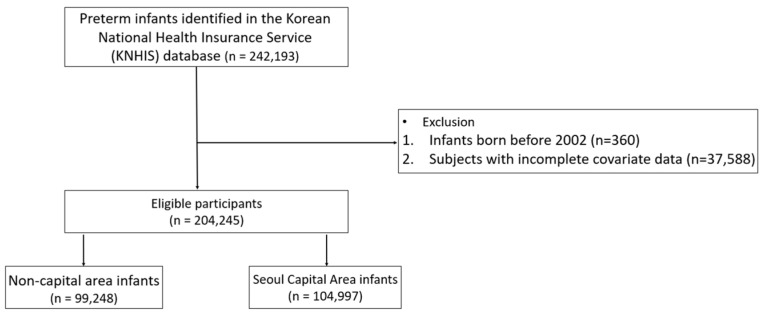
Flow chart of the study design.

In total, 242,193 preterm infants were identified from the KNHIS database. After excluding 360 infants born before 1 January 2002, and 37,588 infants with incomplete demographic or clinical information, the final analytic sample comprised 204,245 preterm infants. These infants were classified according to their parents’ registered residential region at birth into the SCA and non-SCA regions. Of the total cohort, 99,248 infants (48.6%) resided in non-SCA regions and 104,997 (51.4%) in the SCA (Figure 1). Baseline characteristics by residential region are summarized in Table 1. Sex distribution was similar across regions, with male infants accounting for 54.9% of births in non-SCA regions and 54.6% in the SCA (*p* = 0.128). Birth weight distributions differed modestly: normal birth weight (NBW) infants accounted for 45.6% of births in non-SCA regions and 47.7% in the SCA, while the proportion of very low birth weight (VLBW) infants was 9.8% and 9.3%, respectively. Early postnatal transfer within one day of birth occurred more frequently in non-SCA regions than in the SCA (5.9% vs. 4.7%). Medical Aid enrollment was also more common in non-SCA regions (0.6% vs. 0.2%). Mothers of infants in non-SCA regions were slightly younger (mean age 32.4 years [SD 4.5] vs. 33.0 years [SD 4.3]) and had fewer antenatal visits (mean 13.1 [SD 7.4] vs. 13.7 [SD 7.6]). Twenty-eight-day mortality was higher among infants residing in non-SCA regions than in the SCA (0.7% vs. 0.5%), and this difference persisted at one year (2.1% vs. 1.7%). With the exception of sex, all between-group differences were statistically significant (*p* < 0.0001).

As summarized in Table 2, preterm infants residing in non-SCA regions had significantly higher risks of both 28-day and 1-year mortality compared with those residing in the SCA, after adjustment for transfer status, medical aid enrollment, maternal age, and number of prenatal visits (28-day mortality: adjusted odds ratio (OR), 1.46; 95% confidence interval (CI), 1.30–1.64; 1-year mortality: adjusted OR, 1.25; 95% CI, 1.17–1.34). When stratified by birth weight category, the disparities were not statistically significant among NBW infants for either 28-day (OR, 1.14; 95% CI, 0.92–1.41; *p* = 0.2421) or 1-year mortality (OR, 1.09; 95% CI, 0.97–1.23; *p* = 0.1576). However, progressively greater disparities emerged in lower birth weight strata. For LBW infants, the adjusted ORs were 1.36 for 28-day mortality (95% CI, 1.19–1.57) and 1.16 for 1-year mortality (95% CI, 1.07–1.25). Among VLBW infants, the adjusted ORs were 1.38 for 28-day mortality (95% CI, 1.18–1.62) and 1.27 for 1-year mortality (95% CI, 1.15–1.41). The largest disparities were observed in ELBW infants, with adjusted ORs of 1.67 for 28-day mortality (95% CI, 1.40–1.97) and 1.54 for 1-year mortality (95% CI, 1.37–1.73). Across the two study periods (Supplementary Tables S1 and S2), mortality disparities persisted for all preterm infants, the adjusted OR for 1-year mortality in non–SCA regions increased from 1.18 (95% CI 1.07–1.31) in 2002–2011 to 1.30 (95% CI 1.19–1.43) in 2012–2021, whereas the adjusted OR for 28-day mortality decreased from 1.62 (95% CI 1.31–2.00) to 1.32 (95% CI 1.14–1.52). Disparities remained non-significant in the NBW group but were consistently larger in lower birth weight strata, particularly in VLBW and ELBW infants.

As shown in Table 3 and Table 4, when infants residing in and receiving their initial treatment within the SCA were used as the reference group, those residing in non-SCA regions but receiving initial treatment in the SCA had significantly higher adjusted odds of mortality (28-day: adjusted OR, 1.53; 95% CI, 1.19–1.97; 1-year: adjusted OR, 1.46; 95% CI, 1.26–1.71). Mortality was also elevated in infants residing and receiving initial treatment in non-SCA regions (28-day: OR, 1.45; 95% CI, 1.28–1.64; 1-year: OR, 1.24; 95% CI, 1.16–1.34). Birth weight-stratified analyses revealed heterogeneous patterns. In the NBW group, disparities were pronounced for infants residing in non-SCA regions but receiving initial treatment in the SCA (28-day: OR, 2.98; 95% CI, 2.05–4.34; 1-year: OR, 2.22; 95% CI, 1.74–2.84), whereas those residing and receiving initial treatment in non-SCA regions showed no significant difference. For LBW infants, both non-SCA residence/SCA treatment and non-SCA residence/non-SCA treatment groups had elevated mortality, with larger effect sizes for 28-day mortality. Importantly, among VLBW and ELBW infants residing in non-SCA regions, initial treatment in SCA hospitals was not associated with increased mortality, whereas initial treatment in non-SCA hospitals was associated with significantly higher mortality. In the VLBW group, non-SCA residence/SCA treatment was not associated with higher mortality (28-day: OR, 1.04; 95% CI, 0.72–1.51; 1-year: OR, 1.03; 95% CI, 0.81–1.32), whereas non-SCA residence/non-SCA treatment showed significantly elevated mortality (28-day: OR, 1.42; 95% CI, 1.21–1.67; 1-year: OR, 1.32; 95% CI, 1.18–1.46). Similarly, among ELBW infants, mortality was not increased for non-SCA residents treated in the SCA (28-day: OR, 0.91; 95% CI, 0.61–1.35; 1-year: OR, 0.92; 95% CI, 0.70–1.20), but was highest for those residing and treated in non-SCA regions (28-day: OR, 1.78; 95% CI, 1.49–2.12; 1-year: OR, 1.68; 95% CI, 1.48–1.89).

Across all preterm infants, treatment in non-SCA hospitals was associated with lower hazards for IVH (hazard ratio (HR), 0.87; 95% CI, 0.82–0.92), NEC (HR, 0.87; 95% CI, 0.80–0.94), ROP (HR, 0.92; 95% CI, 0.90–0.93), and BPD (HR, 0.77; 95% CI, 0.74–0.80), but higher hazards for late-onset sepsis (HR, 1.17; 95% CI, 1.07–1.27) compared with SCA hospitals (Table 5). In VLBW and ELBW groups, the protective association for IVH observed in higher birth weight groups was attenuated or reversed, with ELBW infants in non-SCA hospitals and non-SCA birth regions showing higher IVH hazards, while late-onset sepsis hazards in these lower birth weight strata remained elevated. As a sensitivity analysis accounting for the competing risk of death, analyses using composite endpoints showed generally higher event hazards in non-SCA hospitals, while the overall pattern of associations across birth weight strata remained similar (Supplementary Table S3).

## 4. Discussion

In this nationwide cohort study (2002–2021), we found that preterm infants residing in non-SCA regions experienced consistently poorer survival than those residing in the SCA, with the disparity becoming progressively larger as birth weight decreased. This graded pattern suggests that the adverse consequences of regional centralization of specialized neonatal care are concentrated among the most clinically fragile infants, who are most dependent on timely access to high-acuity perinatal and neonatal services. These findings highlight the need to strengthen regionalized systems of perinatal care—both by improving local capacity in non-SCA regions and by ensuring rapid access pathways to tertiary centers for the highest-risk pregnancies and newborns—so that place of residence does not determine survival for extremely vulnerable infants.

Our results align with previous studies reporting higher neonatal mortality in underserved regions of Korea [14,18,19], and extend them by leveraging a 20-year, population-level claims database. The graded increase in ORs across weight strata mirrors international reports showing that very low and extremely low birth weight infants are especially sensitive to variations in NICU capability and promptness of care [8,14,26,27]. Unlike prior work limited to initial hospitalization outcomes, our one-year follow-up captures both in-hospital and post-discharge mortality, thus reflecting the combined performance of tertiary centers and community health services.

Overall, our birth weight-stratified results suggest different mechanisms by risk group. For ELBW/VLBW infants, the largest disparities appear consistent with differential access to high-acuity neonatal intensive care and delays in time-critical interventions in non-SCA regions, whereas these differences were attenuated when care occurred in the SCA. In contrast, the elevated mortality observed among NBW/LBW infants receiving initial treatment in the SCA likely reflects a combination of selective referral of clinically complex cases and potential risks arising from delayed local stabilization or delayed initiation of definitive care. One plausible mechanism contributing to the observed disparities is that, outside of regional referral centers, access to Level 3 NICUs and experienced neonatal staff in non-SCA regions may be more limited, which could delay critical interventions for ELBW and VLBW infants [14,16,18,19]. Consistent with this mechanism, we observed no significant excess risk among ELBW and VLBW infants transferred to and treated in the SCA, whereas mortality was markedly higher when infants both resided and received care in non-SCA regions. International comparisons underscore the importance of such system-level capacity: Japan, for example, has one of the lowest neonatal mortality rates worldwide, particularly among extremely preterm infants. This advantage is attributed to the concentration of resources in perinatal medical centers with integrated maternal–fetal and neonatal intensive care, structured antenatal transfer systems, and specialized management protocols. Reflecting these practices, Japan reports lower incidences of IVH, NEC, and late-onset sepsis, but higher rates of ROP and BPD. Our findings showed a similar pattern: SCA hospitals were associated with lower hazards of IVH and late-onset sepsis among smaller infants, whereas the risk of ROP was higher in the SCA. As in Japan, this likely reflects improved survival among the most vulnerable infants, with longer survival increasing the opportunity for ROP to develop [23]. From a policy perspective, our findings underscore the need for a two-pronged strategy for resource allocation to advance regionalization and achieve self-sufficient neonatal care in non-SCA regions. First, our birth weight-stratified analyses (Table 3 and Table 4) indicate that, among VLBW/ELBW infants residing in non-SCA regions, mortality was lower when initial treatment occurred in SCA hospitals than when it occurred in non-SCA hospitals. Although transfer timing cannot be directly determined in claims data, this pattern suggests that ensuring timely access to higher-level perinatal/neonatal care—potentially through antenatal maternal transfer to tertiary centers—may be preferable to postnatal neonatal transfer for critically high-risk preterm infants. Accordingly, to improve survival among ELBW/VLBW infants, resources should be strategically consolidated to strengthen high-acuity NICU care capability in non-SCA regions. This includes prioritizing the recruitment, training, and retention of dedicated neonatal intensivists and other highly experienced pediatric specialists, as well as developing the infrastructure and protocols required to deliver Level 3-equivalent care. These efforts may enable management without transfer to the SCA and reduce early transfers and overall mortality. Second, for NBW/LBW infants, our residence–treatment analysis showed that receiving initial treatment outside their residential region, specifically in the SCA, was paradoxically associated with higher mortality. The higher mortality observed in this group may partly reflect selection processes, whereby NBW infants who received their initial treatment in the SCA are more likely to represent clinically complex or deteriorating cases, including those with suspected congenital anomalies, antenatal complications, or acute perinatal events that prompted urgent referral or transfer. However, such residual confounding is unlikely to fully explain the observed association. For the 28-day mortality observed among normal birth weight infants (OR = 2.98; 95% CI, 2.05–4.34), the corresponding E value (5.4 for the point estimate and 3.5 for the lower confidence bound) indicates that an unmeasured confounder would need to be associated with both treatment location and mortality by a risk ratio of at least 3.5 to 5.4 to nullify the effect an implausibly strong confounding structure given the extensive adjustment for birth weight, antenatal visits, transfer status, and socioeconomic factors [28]. Moreover, the finding that mortality disparities were most pronounced among normal birth weight infants, while attenuated among lower birth weight strata, supports the interpretation that delayed access to initial treatment, rather than baseline clinical severity alone, may underlie the observed patterns. Taken together, these findings suggest that a substantial subset of NBW/LBW infants could be safely managed locally with comparatively modest resources and that failure to receive timely treatment within their residential region may introduce avoidable risks, such as delays in stabilization or deterioration before treatment initiation. Accordingly, prioritizing standardized local stabilization and definitive care for NBW/LBW infants, while reserving transfers for clearly defined high acuity indications, may yield immediate survival gains without large capital investments.

This study’s strengths include nationwide representativeness of infants who were clinically diagnosed with and received inpatient treatment for preterm birth, with linked maternal information, based on a large, nationally representative claims dataset spanning 2002–2021. Nonetheless, several limitations warrant consideration. We lacked several important covariates that could confound the association between region and mortality, including detailed clinical information (e.g., gestational age in completed weeks, Apgar scores, and severity-of-illness markers) and key sociodemographic and behavioral factors (e.g., marital status, smoking, and alcohol use). Although we used insurance eligibility/coverage category as a proxy for socioeconomic position, residual confounding may remain. In addition, fetal growth restriction was only incompletely captured through maternal claims diagnoses, and small for gestational age may be under-coded or inconsistently recorded in claims data; moreover, we could not reliably ascertain plurality (singleton vs. multiple gestations) across the full study period. We therefore did not incorporate these variables due to concerns about misclassification, which may further contribute to residual confounding. Within a given birth weight category, gestational age reflects both physiologic immaturity (lower gestational age) and the possibility of fetal growth restriction (higher gestational age at the same birth weight); therefore, residual confounding due to unmeasured gestational age may not be unidirectional. Coding errors inherent to claims data and potential unmeasured socioeconomic factors (e.g., maternal education, urbanicity) could also bias estimates. To mitigate this limitation, we used diagnosis-based proxies to classify prematurity and birth weight (NBW/LBW/VLBW/ELBW via KCD codes) and adjusted for maternal age, number of antenatal visits, transfer status, and Medical Aid enrollment. Although diagnosis-based measures are imperfect, coding standards and the claims submission system are uniform across regions; moreover, in Korea, National Health Insurance coverage is near-universal and financial support (reduced cost-sharing) is available for preterm and low birth weight infants who require hospital-based medical care (e.g., <37 weeks’ gestation or birth weight ≤ 2500 g), which likely incentivizes complete diagnostic coding when preterm-related care is provided. Therefore, any misclassification or exclusion (e.g., infants with a low birth weight code but without a preterm diagnosis) is unlikely to be strongly differential by region and would, if anything, bias estimates toward the null. Accordingly, the observed regional differences are unlikely to be explained solely by unmeasured clinical detail. Beyond potential coding errors, some infants with relatively mild symptoms near term who did not receive medical care may not have been included in the claims data; thus, our cohort should be interpreted as medically attended preterm infants requiring medical intervention captured in claims, rather than all preterm births in the general population. Nevertheless, these infants are somewhat outside the focus of this study, which centers on regional disparities in preterm intensive care, and would be more appropriately examined in a separate line of research. Additional neonatal outcomes (e.g., NICU admission, Apgar scores, and length of stay) were not evaluated because they are not available or not reliably captured as clinical outcomes in administrative claims data, and future studies using richer clinical datasets should assess these intermediate outcomes alongside mortality. Finally, although regions were defined by residence at birth, some infants received care across multiple jurisdictions, complicating attribution of outcomes to specific health systems. Moreover, our region definition is based on administrative residence and may not perfectly map onto functional neonatal care networks; therefore, regional disparities should not be interpreted as purely reflecting differences between health systems.

## 5. Conclusions

Our population-based analysis reveals significant regional inequities in preterm infant survival, most pronounced among the smallest infants. Future research should incorporate richer clinical detail, such as individual level severity scores, and examine long-term neurodevelopmental outcomes. Ultimately, concerted efforts to bolster both hospital-based and community-based neonatal care in non-SCA regions—supported by strengthened Level III-equivalent NICU capability—are essential to ensure that all Korean preterm infants, regardless of birthplace, enjoy an equal chance at survival and healthy development.

## Figures and Tables

**Table 1:** Patient characteristics of the 204,245 Eligible Individuals, KNHI database 2002–2021.

	Non-Capital Area Infants (N = 99,248)	Seoul Capital Area Infants (N = 104,997)	*p* Value
**Sex**			
Male	54,503 (54.9)	57,308 (54.6)	0.1280
Female	44,745 (45.1)	47,689 (45.4)	
**Birth weight**			
≥2500 g	45,278 (45.6)	50,617 (47.7)	<0.0001
1500–2499 g	44,281 (44.6)	44,487 (42.0)	
1000–1499 g	5748 (5.8)	5115 (4.8)	
<1000 g	3941 (4.0)	4778 (4.5)	
**Early transfer (within 1 day)**	5878 (5.9)	4945 (4.7)	<0.0001
**Medical aid**	577 (0.6)	244 (0.2)	<0.0001
**Maternal age in years, Mean ± SD**	32.4 ± 4.5	33.0 ± 4.3	<0.0001
**Perinatal visits, Mean ± SD**	13.1 ± 7.4	13.7 ± 7.6	<0.0001
**28-day mortality**	695 (0.7)	549 (0.5)	<0.0001
**1-year mortality**	2086 (2.1)	1800 (1.7)	<0.0001

Abbreviations: SD, standard deviation. n (%) unless otherwise noted.

**Table 2:** 28-day and 1-year mortality by residential region: adjusted odds ratios by birth weight category (SCA vs. non-SCA).

Birth Weight Category	28-Day Mortality OR (95% CI)	*p* Value	1-Year Mortality OR (95% CI)	*p* Value
All preterm infants	1.46 (1.30–1.64)	<0.0001	1.25 (1.17–1.34)	<0.0001
≥2500 g	1.14 (0.92–1.41)	0.2421	1.09 (0.97–1.23)	0.1576
1500–2499 g	1.36 (1.19–1.57)	<0.0001	1.16 (1.07–1.25)	0.0003
1000–1499 g	1.38 (1.18–1.62)	<0.0001	1.27 (1.15–1.41)	<0.0001
<1000 g	1.67 (1.40–1.97)	<0.0001	1.54 (1.37–1.73)	<0.0001

Abbreviations: SCA, Seoul Capital Area; OR, odds ratio; CI, confidence interval. Reference group: Infants residing in the SCA. All models adjusted for sex, categorical birth weight group, transfer status, medical aid, maternal age, and prenatal visits; birth weight was excluded in stratified models.

**Table 3:** 28-day mortality by residence–treatment region combination: adjusted odds ratios by birth weight category.

Birth Weight Category	SCA Residence/ SCA Treatment (Ref.)	Non-SCA Residence/ SCA Treatment OR (95% CI)	Non-SCA Residence/ Non-SCA Treatment OR (95% CI)
All preterm infants	1.00	1.53 (1.19–1.97)	1.45 (1.28–1.64)
≥2500 g	1.00	2.98 (2.05–4.34)	0.96 (0.77–1.21)
1500–2499 g	1.00	1.65 (1.20–2.28)	1.32 (1.14–1.52)
1000–1499 g	1.00	1.04 (0.72–1.51)	1.42 (1.21–1.67)
<1000 g	1.00	0.91 (0.61–1.35)	1.78 (1.49–2.12)

Abbreviations: SCA, Seoul Capital Area; OR, odds ratio; CI, confidence interval. All models adjusted for sex, categorical birth weight group, transfer status, medical aid, maternal age, and prenatal visits; birth weight was excluded in stratified models.

**Table 4:** 1-year mortality by residence–treatment region combination: adjusted odds ratios by birth weight category.

Birth Weight Category	SCA Residence/ SCA Treatment (Ref.)	Non-SCA Residence/ SCA Treatment OR (95% CI)	Non-SCA Residence/ Non-SCA Treatment OR (95% CI)
All preterm infants	1.00	1.46 (1.26–1.71)	1.24 (1.16–1.34)
≥2500 g	1.00	2.22 (1.74–2.84)	1.01 (0.90–1.15)
1500–2499 g	1.00	1.61 (1.34–1.94)	1.12 (1.03–1.21)
1000–1499 g	1.00	1.03 (0.81–1.32)	1.32 (1.18–1.46)
<1000 g	1.00	0.92 (0.70–1.20)	1.68 (1.48–1.89)

Abbreviations: SCA, Seoul Capital Area; OR, odds ratio; CI, confidence interval. All models adjusted for sex, categorical birth weight group, transfer status, medical aid, maternal age, and prenatal visits; birth weight was excluded in stratified models.

**Table 5:** Major morbidities by region of initial treatment: adjusted hazard ratios by birth weight category (SCA vs. non-SCA).

Birth Weight Category	IVH HR (95% CI)	NEC HR (95% CI)	Late-Onset Sepsis HR (95% CI)	ROP HR (95% CI)	BPD HR (95% CI)
All preterm infants	0.87 (0.82–0.92)	0.87 (0.80–0.94)	1.17 (1.07–1.27)	0.92 (0.90–0.93)	0.77 (0.74–0.80)
≥2500 g	0.79 (0.71–0.88)	0.91 (0.77–1.08)	0.82 (0.68–0.97)	0.82 (0.79–0.84)	0.58 (0.53–0.71)
1500–2499 g	0.83 (0.78–0.89)	0.75 (0.69–0.83)	1.15 (1.05–1.27)	0.94 (0.93–0.96)	0.68 (0.65–0.71)
1000–1499 g	1.09 (0.99–1.21)	0.80 (0.70–0.91)	1.42 (1.24–1.63)	0.95 (0.91–0.98)	0.81 (0.77–0.85)
<1000 g	1.30 (1.14–1.48)	0.98 (0.84–1.15)	1.48 (1.24–1.77)	0.90 (0.85–0.95)	0.92 (0.86–0.97)

Abbreviations: SCA, Seoul Capital Area; HR, hazard ratio; CI, confidence interval; IVH, intraventricular hemorrhage; NEC, necrotizing enterocolitis; ROP, retinopathy of prematurity; BPD, bronchopulmonary dysplasia. Reference group: Infants initially treated in SCA hospitals. All models adjusted for transfer status, medical aid, maternal age, and prenatal visits; birth weight was excluded in stratified models.

## Data Availability

The data used in this study were obtained from the Korean National Health Insurance Service (KNHIS) of South Korea under a data use agreement; therefore, restrictions apply to their availability. The data are not publicly available and can be accessed only through an application and approval process administered by the KNHIS.

## Supplementary Materials

The following supporting information can be downloaded at: https://www.mdpi.com/article/10.3390/children13020217/s1, Table S1: 28-day mortality by residential region: adjusted odds ratios by birth weight category (SCA vs. non-SCA), stratified by period (2002–2011 vs. 2012–2021); Table S2: 1-year mortality by residential region: adjusted odds ratios by birth weight category (SCA vs. non-SCA), stratified by period (2002–2011 vs. 2012–2021); Table S3: Sensitivity analysis for major morbidities using a composite endpoint (morbidity-or-death) by region of initial treatment: adjusted hazard ratios by birth weight category (SCA vs. non-SCA).

## Abbreviations

The following abbreviations are used in this manuscript:

BPD	Bronchopulmonary Dysplasia
CI	Confidence Interval
ELBW	Extremely Low Birth Weight
HR	Hazard Ratio
ICD	International Classification of Diseases
IVH	Intraventricular Hemorrhage
KCD	Korean Standard Classification of Diseases
LBW	Low Birth Weight
NEC	Necrotizing Enterocolitis
KNHIS	Korean National Health Insurance Service
NICU	Neonatal Intensive Care Unit
NBW	Normal Birth Weight
OR	Odds Ratio
ROP	Retinopathy of Prematurity
SCA	Seoul Capital Area
SD	Standard Deviation
VLBW	Very Low Birth Weight
