# Supplementary material for: Birth Weight-Dependent Regional Disparities in 28-Day and 1-Year Survival of Preterm Infants: Seoul Capital Area vs. Non-Capital Regions, South Korea, 2002–2021
Source: Children (Basel). 2026 Feb 2;13(2):217. doi: 10.3390/children13020217 (PMC12938972; doi:10.3390/children13020217)
Supplement: Supplementary file 1 [file children-13-00217-s001.zip › children-4113143-supplementary.pdf]

Supplementary Tables

Table S1. 28-day mortality by residential region: adjusted odds ratios by birth-weight category (SCA vs non-SCA), stratified by period (2002–2011 vs 2012–2021)

	2002–2011		2012–2021	
**Group**	**Adjusted OR (95% CI)**	***P* value**	**Adjusted OR (95% CI)**	***P* value**
All preterm infants	1.62 (1.31–2.00)	<0.0001	1.32 (1.14–1.52)	0.0002
≥ 2,500g	1.18 (0.84–1.66)	0.3291	1.09 (0.83–1.44)	0.5266
1,500–2,499g	1.54 (1.17–2.01)	0.0019	1.25 (1.06–1.48)	0.0068
1,000–1,499g	1.57 (1.12–2.19)	0.0091	1.32 (1.11–1.58)	0.0021
< 1,000g	2.00 (1.41–2.83)	0.0001	1.44 (1.18–1.75)	0.0003

Abbreviations: SCA, Seoul Capital Area; OR, odds ratio; CI, confidence interval.
Reference group: Infants residing in the SCA.
All models adjusted for sex, categorical birthweight group, transfer status, medical aid, maternal age, and prenatal visits; birth weight was excluded in stratified models.

Table S2. 1-year mortality by residential region: adjusted odds ratios by birth-weight category (SCA vs non-SCA), stratified by period (2002–2011 vs 2012–2021)

	**2002-2011**		**2012-2021**	
**Group**	**Adjusted OR (95% CI)**	***P* value**	**Adjusted OR (95% CI)**	***P* value**
All preterm infants	1.18 (1.07–1.31)	0.0010	1.30 (1.19–1.43)	<0.0001
≥ 2,500g	1.10 (0.93–1.29)	0.2700	1.09 (0.92–1.30)	0.3081
1,500–2,499g	1.03 (0.91–1.14)	0.6674	1.27 (1.14–1.41)	<0.0001
1,000–1,499g	1.15 (0.96–1.39)	0.1356	1.39 (1.22–1.57)	<0.0001
< 1,000g	1.46 (1.20–1.78)	0.0033	1.55 (1.34–1.80)	<0.0001

Abbreviations: SCA, Seoul Capital Area; OR, odds ratio; CI, confidence interval.
Reference group: Infants residing in the SCA.
All models adjusted for sex, categorical birthweight group, transfer status, medical aid, maternal age, and prenatal visits; birth weight was excluded in stratified models.

Table S3. Sensitivity analysis for major morbidities using a composite endpoint (morbidity-or-death) by region of initial treatment: adjusted hazard ratios by birth-weight category (SCA vs non-SCA)

Birthweight category	IVH	NEC	Late-onset Sepsis	ROP	BPD
All preterm infants	0.99 (0.95–1.04)	1.04 (0.99–1.10)	1.25 (1.19–1.32)	0.93 (0.91–0.94)	0.85 (0.82–0.88)
≥ 2,500g	0.85 (0.79–0.92)	0.94 (0.85–1.03)	0.97 (0.96–0.97)	0.82 (0.80–0.85)	0.71 (0.66–0.76)
1,500–2,499g	0.92 (0.87–0.97)	0.91 (0.85–0.97)	1.14 (1.08–1.22)	0.95 (0.93–0.97)	0.74 (0.71–0.76)
1,000–1,499g	1.19 (1.11–1.28)	1.08 (1.00–1.18)	1.41 (1.30–1.53)	0.98 (0.95–1.02)	0.87 (0.84–0.91)
< 1,000g	1.47 (1.35–1.60)	1.37 (1.25–1.51)	1.68 (1.53–1.84)	1.01 (0.96–1.06)	1.03 (0.98–1.09)

Abbreviations: SCA, Seoul Capital Area; HR, hazard ratio; CI, confidence interval; IVH, intraventricular hemorrhage; NEC, necrotizing enterocolitis; ROP, retinopathy of prematurity; BPD, bronchopulmonary dysplasia.

Reference group: Infants initially treated in SCA hospitals.

All models adjusted for sex, categorical birthweight group, transfer status, medical aid, maternal age, and prenatal visits; birth weight was excluded in stratified models.
